# Plant anatomy: The next episode—high throughput sectioning and image processing with AnatomyArray

**DOI:** 10.1093/plcell/koaf228

**Published:** 2025-09-23

**Authors:** Gwendolyn K Kirschner

**Affiliations:** Assistant Features Editor, the Plant Cell, American Society of Plant Biologists; The James Hutton Institute, Invergowrie, Dundee DD2 5DA, UK

Since the 1860s, scientists have used paraffin embedding to prepare tissue samples for sectioning, whereby samples are fixed, dehydrated, infiltrated with a solvent, and embedded in paraffin wax, a petroleum derivative (van der Lem et al. 2021). The embedded samples can then be sectioned using a microtome for microscopic examination of tissue anatomy. This classical method, however, allows for the sectioning of only 1 sample at a time, rendering the analysis of a collection of samples labor intensive and time consuming. In new work, **Yikeng Cheng, Jiawei Shi, and colleagues (Cheng et al. 2025)** present a workflow for high-throughput sectioning of multiple tissues (AnatomyArray) in combination with a fully automated image processing tool (AnatomyNet).

The AnatomyArray is a brass device consisting of a top plate, 2 limit plates (upper and lower), a down plate, and 2 fasteners from top to down (Fig.). The plant samples are placed in sample-anchor traps in the limit plates. The authors designed devices for different plant organs, such as roots, stems, and leaves, which differ in size and number of sample traps in the limit plates. In the case of roots, 1 to 4 root samples can be placed into each sample holder, totaling 144 samples per plate. The entire device with all samples is then subjected to dehydration, clearing, filtration, and paraffin embedding. The device is then disassembled, and the lower limit plate holds the paraffin block for sectioning. This block can be used for microtome sectioning, yielding multiple glass slides with the tissue sections. These can be dewaxed and scanned with a high-speed fluorescent scanner, which can detect cell wall autofluorescence (Fig.). The slides can also be used for the detection of nucleic acids via *in situ* hybridization and proteins via immunohistochemistry. Since the sections are consecutive, it is also possible to reconstruct the tissue in 3D.

**Figure.**
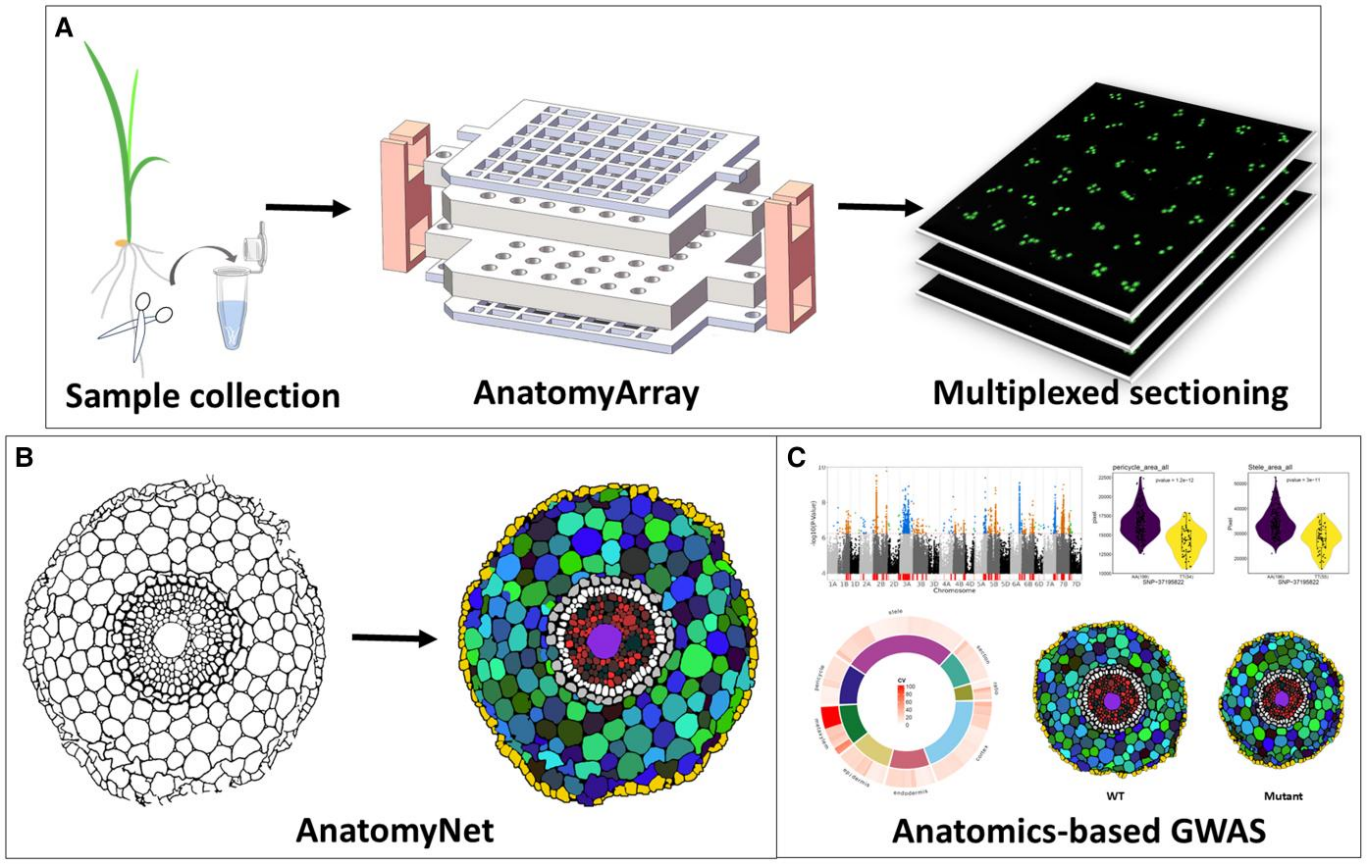
Pipeline for anatomy-based genetic studies using the AnatomyArray system. **A)** The AnatomyArray system can be used for multiplexed tissue processing and sectioning. **B)** The AnatomyNet software provides fully automated image analysis. **C)** Application of the workflow for anatomy-based genome-wide association studies. Adapted from Cheng et al. (2025), Figure 6.

For image processing, the authors developed the software “AnatomyNet,” which first uses a deep learning–based semantic segmentation method for automated identification, extraction, and quantification of anatomical features at the cellular level (Fig.). Subsequently, the software can quantify in total 212 anatomical traits, including the size, number, shape, dimensions, location, and distribution of cells and tissues. Comparing the software with other traditionally used processing and segmentation methods showed that AnatomyNet had the highest correlation to manual measurements.

The authors applied their workflow to phenotype root anatomy in a panel of over 350 wheat germplasms, integrating their data with genome-wide association studies (Fig.). This led to the identification of more than 90 quantitative trait loci (QTLs) across traits of all root tissues. Some QTLs were shared among multiple tissues, suggesting that anatomical structure is shaped by a combination of traits in different tissues. One candidate gene, *SQUAMOSA PROMOTER BINDING PROTEIN-LIKE 14* (*TaSPL14*), was associated with stele and pericycle size and cell number. By mutant analysis, transcriptome analysis, and hormonal profiling, the authors demonstrated that *TaSPL14* regulates root tissue size by coordinating anatomical traits across tissues, likely by maintaining hormone homeostasis.

In summary, this study presents an accessible, high-throughput tissue sectioning and image analysis pipeline that enables detailed investigation of plant tissue anatomy. By making the models and software publicly available, the authors support broad adoption and ongoing improvement of segmentation accuracy across diverse plant tissues and species.

## Recent related articles in *The Plant Cell*

- Liu et al. (2023) analyzed how SHORT ROOT, INDETERMINATE DOMAIN, and PIN-FORMED (PIN) family members regulate leaf anatomy in rice and green millet.
- Hu et al. (2024) established a large-volume, fully automated cell reconstruction to create a 3D cell atlas of different plant tissues.
